# Genomic and Phenotypic Trait Variation of the Opportunistic Human Pathogen Aspergillus flavus and Its Close Relatives

**DOI:** 10.1128/spectrum.03069-22

**Published:** 2022-11-01

**Authors:** E. Anne Hatmaker, Manuel Rangel-Grimaldo, Huzefa A. Raja, Hadi Pourhadi, Sonja L. Knowles, Kevin Fuller, Emily M. Adams, Jorge D. Lightfoot, Rafael W. Bastos, Gustavo H. Goldman, Nicholas H. Oberlies, Antonis Rokas

**Affiliations:** a Department of Biological Sciences, Vanderbilt University, Nashville, Tennessee, USA; b Evolutionary Studies Initiative, Vanderbilt University, Nashville, Tennessee, USA; c Department of Chemistry & Biochemistry, University of North Carolina at Greensboro, Greensboro, North Carolina, USA; d Department of Microbiology and Immunology, University of Oklahoma Health Science Center, Oklahoma City, Oklahoma, USA; e Biosciences Center, Federal University of Rio Grande do Norte, Natal, Brazil; f Faculdade de Ciências Farmacêuticas de Ribeirão Preto, Universidade de São Paulo, Ribeirão Preto, Brazil; Institut Pasteur

**Keywords:** *Aspergillus flavus*, genomes, phenotypic variation, secondary metabolites, fungal keratitis, aspergillosis, biosynthetic gene cluster, comparative genomics, evolution, genomics, pathogenicity, secondary metabolism

## Abstract

Fungal diseases affect millions of humans annually, yet fungal pathogens remain understudied. The mold Aspergillus flavus can cause both aspergillosis and fungal keratitis infections, but closely related species are not considered clinically relevant. To study the evolution of A. flavus pathogenicity, we examined genomic and phenotypic traits of two strains of A. flavus and three closely related species, Aspergillus arachidicola (two strains), Aspergillus parasiticus (two strains), and Aspergillus nomiae (one strain). We identified >3,000 orthologous proteins unique to A. flavus, including seven biosynthetic gene clusters present in A. flavus strains and absent in the three nonpathogens. We characterized secondary metabolite production for all seven strains under two clinically relevant conditions, temperature and salt concentration. Temperature impacted metabolite production in all species, whereas salinity did not affect production of any species. Strains of the same species produced different metabolites. Growth under stress conditions revealed additional heterogeneity within species. Using the invertebrate fungal disease model Galleria mellonella, we found virulence of strains of the same species varied widely; A. flavus strains were not more virulent than strains of the nonpathogens. In a murine model of fungal keratitis, we observed significantly lower disease severity and corneal thickness for A. arachidicola compared to other species at 48 h postinfection, but not at 72 h. Our work identifies variations in key phenotypic, chemical, and genomic attributes between A. flavus and its nonpathogenic relatives and reveals extensive strain heterogeneity in virulence that does not correspond to the currently established clinical relevance of these species.

**IMPORTANCE** Aspergillus flavus is a filamentous fungus that causes opportunistic human infections, such as aspergillosis and fungal keratitis, but its close relatives are considered nonpathogenic. To begin understanding how this difference in pathogenicity evolved, we characterized variation in infection-relevant genomic, chemical, and phenotypic traits between strains of A. flavus and its relatives. We found extensive variation (or strain heterogeneity) within the pathogenic A. flavus as well as within its close relatives, suggesting that strain-level differences may play a major role in the ability of these fungi to cause disease. Surprisingly, we also found that the virulence of strains from species not considered to be pathogens was similar to that of A. flavus in both invertebrate and murine models of disease. These results contrast with previous studies on Aspergillus fumigatus, another major pathogen in the genus, for which significant differences in infection-relevant chemical and phenotypic traits are observed between closely related pathogenic and nonpathogenic species.

## INTRODUCTION

Fungal infections affect millions of people worldwide annually but remain understudied and poorly understood (1). Severe fungal infections in humans, such as aspergillosis, commonly affect immunocompromised individuals (2). Invasive aspergillosis is an umbrella term describing a range of respiratory infections caused by inhalation of asexual spores of several Aspergillus species that grow into human tissue (3). Around 300,000 cases of invasive aspergillosis are identified each year, with a high mortality rate (2). Another form of aspergillosis, chronic pulmonary aspergillosis, impacts ~3 million people annually (2). Invasive aspergillosis and chronic pulmonary aspergillosis can also co-occur in immunocompetent patients with severe viral infections, such as severe acute respiratory syndrome coronavirus 2 (4) and influenza virus (5).

Fungal keratitis, or inflammation of the cornea due to a fungal infection, affects otherwise-healthy individuals, although immunocompromised individuals have higher rates of infection than immunocompetent ones (2). Globally, at least 1,000,000 cases of fungal keratitis occur annually, including both yeast and filamentous fungal infections (2), over 15,000 of which are in the United States, and ~10% of cases result in eye removal due to late diagnosis or poor therapeutic outcomes (6). Fungal keratitis can lead to blindness, and affected individuals are typically infected through small wounds on the eye’s surface (7). The disease primarily afflicts outdoor workers (e.g., farmers) and contact lens wearers (8, 9). Sand and vegetative material often cause the initial wounds through which the infection spreads (10). In western countries, including in the United States, contact lens use is the primary risk factor (11). The Global Action Fund for Fungal Infections has designated fungal keratitis a public health priority (12). Childhood fungal keratitis is the most frequent cause of corneal blindness worldwide (13), which occurs in 40% of severe fungal keratitis cases (12).

Aspergillus flavus, an opportunistic human pathogen, is estimated to cause 10% of invasive aspergillosis infections worldwide, second only to Aspergillus fumigatus, and up to 80% of keratitis infections from Aspergillus species (14). Retrospective studies from Cuba and Pakistan identified more cases of chronic pulmonary aspergillosis caused by A. flavus than other Aspergillus species (15, 16). A. flavus is widespread in the environment, particularly in tropical regions (17), but we do not currently know which A. flavus traits impact virulence in humans. The taxonomic section *Flavi* includes A. flavus and closely related species such as Aspergillus parasiticus, Aspergillus arachidicola, and Aspergillus nomiae (18). Species in section *Flavi* share several morphological characteristics (19), including production of the carcinogen aflatoxin (18), but do not cause human disease at similar rates (20). Throughout this report, we use “nonpathogenic” to refer to species rarely or never isolated from human or animal infections and which are not considered clinically relevant.

A. flavus is more commonly isolated in invasive aspergillosis and fungal keratitis cases than its close relative *A. parasiticus* (17). Okun et al. (21) observed similar geographic ranges for the two species in agricultural fields in Kenya, although A. flavus was isolated about twice as often (63% of all isolates were A. flavus versus 28% A. parasiticus). In the western United States, prevalence and occurrence density of the two species varied based on location, with 48% of Aspergillus isolates identified as *A. parasiticus* at some sampling locations (22). Despite the prevalence of *A. parasiticus* in the environment, the species is rarely isolated from patients, deviating from expectations based on species distribution and density data, indicating additional factors may be necessary for human pathogenicity. We note that *A. parasiticus* has been occasionally identified as a causal agent of human infections, but these infections occur at a much lower rate than those caused by A. flavus, and we were unable to identify published case studies.

A. flavus and *A. parasiticus* also both grow well at 37°C and 42°C (23); thermotolerance is considered key for Aspergillus pathogenicity in general (24, 25). Growth at 37°C has also been shown to change the metabolic profile of Aspergillus species (26). Secondary metabolites, which are small organic molecules with potent bioactivities, enable fungi to augment their environment with defensive compounds (27) and can impact virulence by modulating host biology (28, 29), as with gliotoxin in A. fumigatus (30). A. flavus and other section *Flavi* species have an even larger arsenal of secondary metabolites than A. fumigatus (31). Temperature can also impact secondary metabolite production. At 37°C, A. flavus produces diverse secondary metabolites (32). At lower temperatures, the most notable A. flavus secondary metabolite is the mycotoxin aflatoxin, a carcinogen (32), which is also produced by several other section *Flavi* species, including *A. parasiticus* (18). A. flavus is also predicted to produce scores of other secondary metabolites (33) which may play a role in human infection.

Current knowledge of A. flavus molecular mechanisms involved in human virulence relies heavily on extrapolation from studies of an A. fumigatus mouse models of both fungal keratitis (34) and respiratory infections (35) rather than direct studies of A. flavus, despite extensive genomic and phenotypic differences between the two species. Although in the same genus, A. flavus and A. fumigatus are not close relatives and are in distinct taxonomic sections of Aspergillus, sections *Flavi* and *Fumigati*, respectively (18); at the level of genome sequence divergence, the two species are as similar to each other as humans are to fish (36). Compared to A. fumigatus, section *Flavi* species have, on average, larger genomes, encode more genes, and are predicted to contain more biosynthetic gene clusters (BGCs) involved in the biosynthesis of secondary metabolites (37). Differences between A. flavus and A. fumigatus include resistance to antifungal drugs, as azole resistance differs between the two species, with triazole resistance considered rare in A. flavus despite comparable exposure to environmental fungicides as A. fumigatus (38). Additional studies investigating genetic determinants of virulence specific to A. flavus, which enable the species to infect humans at a higher rate than closely related species, as well as the drug resistance profiles of diverse strains and species, are needed to better understand the pathogenicity of A. flavus.

To begin addressing the evolution of pathogenicity in section *Flavi*, we examined the genomes, secondary metabolite profiles, and other infection-relevant phenotypic traits (e.g., virulence) of two A. flavus strains and strains from three closely related species that rarely infect humans: *A. arachidicola* (two strains), *A. parasiticus* (two strains), and *A. nomiae* (one strain). The species were chosen based on their phylogenetic placement within the section and close evolutionary affinity, their difference in clinical relevance, and shared characteristics with A. flavus, such as aflatoxin production. We sequenced seven strains from the four species and identified proteins unique to A. flavus strains, including BGCs absent in the nonpathogenic species. Temperature impacted secondary metabolite production in all four species, but secondary metabolites unique to A. flavus were not identified under these conditions. Antifungal drug resistance did not differ appreciably between strains or species, but strains of the same species exhibited variation in growth under certain stress conditions. Evaluation of virulence using an invertebrate model of disease revealed additional variations between strains of the same species, and A. flavus strains were not the most virulent in this model. Virulence in a murine model of fungal keratitis was tested with one strain each from A. flavus, *A. parasiticus*, and *A. arachidicola*, along with a reference strain of A. fumigatus, revealing significantly lower disease severity and corneal thickness in mice infected with *A. arachidicola* compared to those infected with the other species. The combination of genomic and phenotypic comparisons revealed similarities and differences between A. flavus and three nonpathogenic close relatives within Aspergillus section *Flavi*. This study provides key data on the genomic, chemical, and phenotypic diversity of closely related pathogenic and nonpathogenic species in Aspergillus section *Flavi* that further our knowledge of how some of these fungi can opportunistically infect humans.

## RESULTS

### Draft genomes for seven strains of A. flavus and nonpathogenic close relatives.

We sequenced and assembled seven genomes from four species with high coverage (81× to 230×). The A. flavus NRRL 1957 draft genome consisted of the fewest scaffolds (143), whereas the draft genome of *A. arachidicola* IC26646 had the most (1,440). *A. nomiae* NRRL 6108 had the smallest genome, at 36.8 Mbp, and *A. parasiticus* NRRL 502 had the largest, at 41.4 Mbp; A. flavus strains had smaller genomes than strains of *A. parasiticus* or *A. arachidicola* (Table 1). All genomes had over 93% of the expected universal single-copy orthologs (Table 2).

**TABLE 1:** Genome sequencing, assembly, and annotation for the seven strains of four Aspergillus species revealed expected genome size and good coverage depth

Species	Strain	Total paired reads	Trimmed paired reads	No. of scaffolds	*N*_50_	Coverage	Size (Mbp)	No. of predicted proteins
Aspergillus arachidicola	IC26645	30,016,585	28,186,702	805	1,139,563	102×	41.2	13,710
Aspergillus *arachidicola*	IC26646	32,284,138	29,911,439	1,440	1,298,300	109×	40.9	13,731
Aspergillus flavus	NRRL 1957	44,750,116	40,985,045	143	1,892,429	163×	37.6	14,216
Aspergillus flavus	NRRL 501	30,987,721	28,845,216	563	1,420,800	111×	38.8	13,997
Aspergillus *nomiae*	NRRL 6108	25,466,314	23,606,091	252	1,660,546	96×	36.9	11,820
Aspergillus parasiticus	NRRL 502	24,439,029	22,469,327	702	95,7843	81×	41.5	13,470
Aspergillus parasiticus	NRRL 2999	63,194,489	62,404,154	516	1,838,014	230×	40.6	13,590

**TABLE 2:** Genome completeness assessment of draft Aspergillus genomes sequenced for this study

Species	Strain	No. of BUSCOs^*a*^				
		Present (%)	Complete	Duplicated	Fragmented	Missing
Aspergillus *arachidicola*	IC26645	97.1	4,072	14	40	79
Aspergillus *arachidicola*	IC26646	97.0	4,065	14	41	85
Aspergillus flavus	NRRL 1957	95.9	4,023	48	53	115
Aspergillus flavus	NRRL 501	96.0	4,023	19	52	116
Aspergillus *nomiae*	NRRL 6108	93.1	3,607	16	158	410
Aspergillus parasiticus	NRRL 502	97.8	4,099	15	21	71
Aspergillus parasiticus	NRRL 2999	98.0	4,105	15	21	65

a BUSCO, benchmarking universal single-copy orthologs.

### Placement of newly sequenced genomes consistent with Aspergillus section *Flavi* phylogeny.

**FIG 1:**
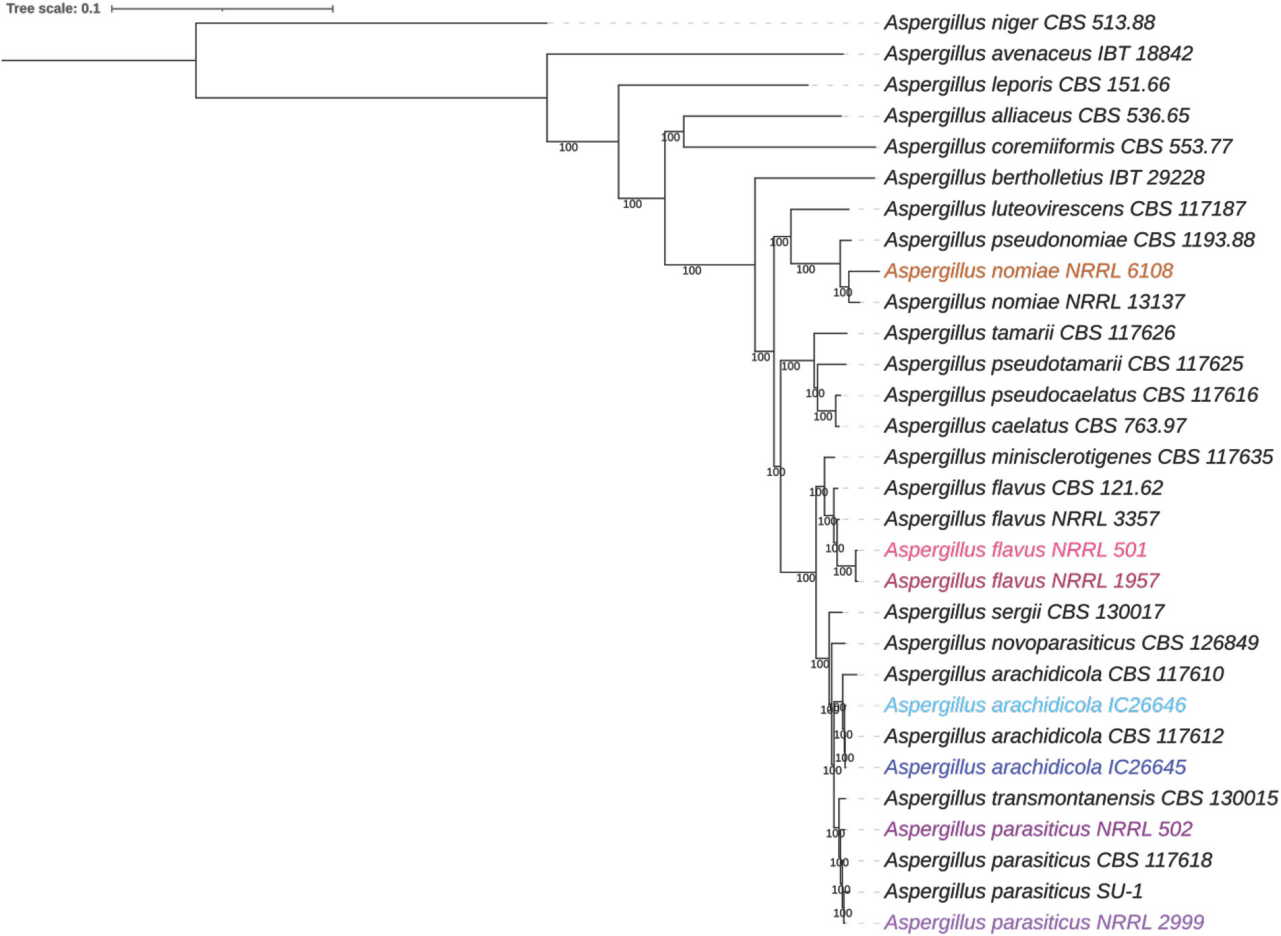
The taxonomic identity of newly sequenced strains was consistent with Aspergillus section *Flavi* phylogeny. A maximum likelihood phylogeny was constructed using 2,422 single-copy orthologs from 30 strains of 20 Aspergillus species, with A. niger as the outgroup. Additional information about strains used can be found in Table 5. Numbers near branches are bootstrap values calculated from 1,000 replicates. Strains sequenced as part of this study are highlighted, with *A. nomiae* NRRL 6108 in orange, A. flavus in pink (NRRL 501 in light pink, NRRL 1957 in dark pink), *A. arachidicola* in blue (IC26645 in dark blue, IC26646 in light blue), and *A. parasiticus* in purple (NRRL 502 in dark purple, NRRL 2999 in light purple).

Our maximum likelihood species tree was built from 2,422 orthologs shared among 20 Aspergillus species (Fig. 1). Species identification was confirmed for all the newly sequenced strains (NRRL 501, 502, 1957, 2999, and 6108 and IC26645 and IC26646) with high support (bootstrap replicates = 100), with each strain placed among the representative strains of the expected species (Table 1). We noted that previous whole-genome sequencing of a strain labeled NRRL 2999 (GCA_012897115.1) was recently determined to be A. flavus rather than *A. parasiticus* (39) and was acknowledged as a clonal derivative of A. flavus NRRL 3357 (40). Our NRRL 2999 strain and *A. parasiticus* SU-1 share 99.98% average nucleotide identity, confirming that our strain, obtained from the NRRL culture collection, is *A. parasiticus*.

### More than 3,000 protein families are unique to the pathogen A. flavus.

**FIG 2:**
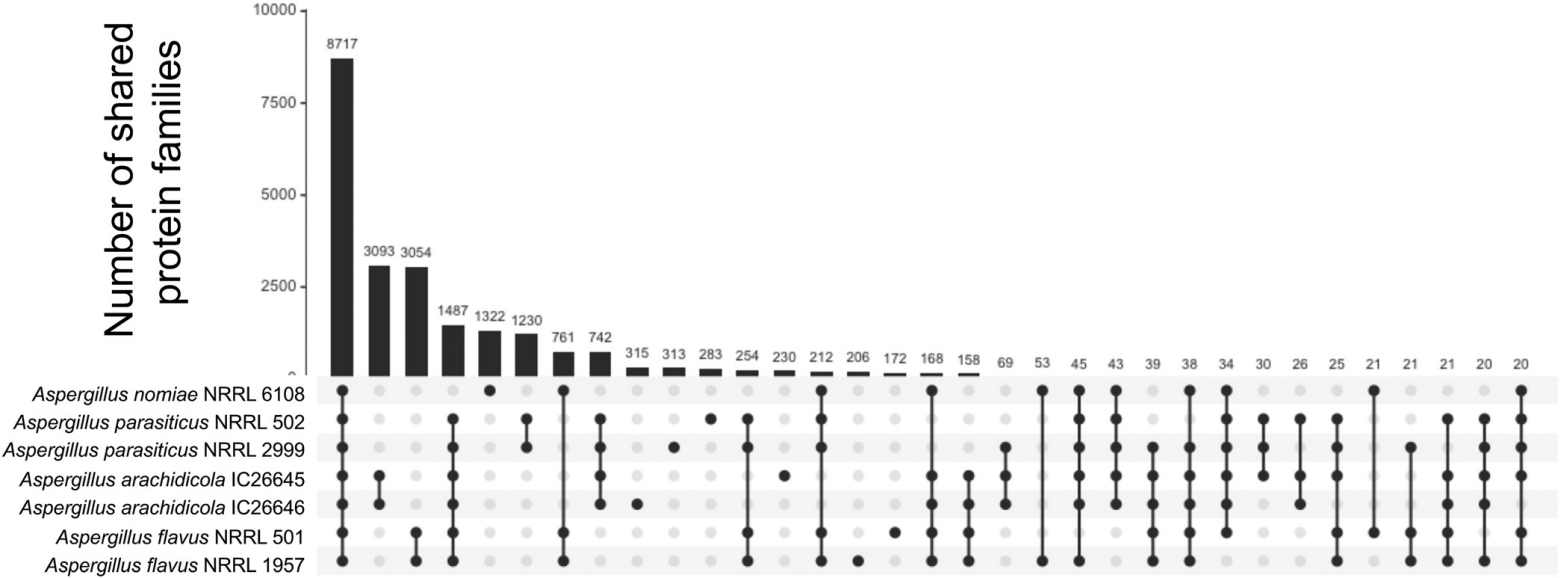
Strains of the same species did not differ substantially in number of predicted protein families present in their genomes. Upset plot shows the number of shared protein families for orthogroups with at least 20 proteins. Linked black circles under the bar plot indicate strains sharing orthologous protein families. Numbers above bars indicate exact numbers of shared families.

We identified 8,717 protein families shared by all seven newly sequenced strains (Fig. 2). A further 3,054 protein families were present in both A. flavus strains but absent from the other five strains. This group of protein families was enriched for the gene ontology (GO) terms GO:0016705 oxidoreductase activity (15 protein families; *P* < 0.0001, hypergeometric distribution) and GO:0055085 transmembrane transport (30 protein families; *P* < 0.0001). *A. arachidicola* strains shared 3,097 unique protein families enriched for GO term GO:0016114 terpenoid biosynthetic process (10 protein families; *P* < 0.00001), and *A. parasiticus* strains shared 1,230 unique protein families without any GO term enrichment. No GO terms were enriched within the 1,322 protein families unique to *A. nomiae*.

We also examined the number of orthogroups (predicted protein families) shared among and within species for all 20 species and 30 strains by using the OrthoFinder results. All 20 species, including the outgroup Aspergillus niger, shared 3,861 orthogroups, and 388 orthogroups were in all section *Flavi* species but absent in A. niger (see Fig. S1 in our supplementary information, available on the FigShare website [https://doi.org/10.6084/m9.figshare.20256336]). The number of orthogroups unique to any particular strain ranged from 229 to 281 (see Fig. S1), which was consistent with our analysis of the 7 strains phenotyped in this study. For example, 280 strain-specific orthogroups were identified for *A. parasiticus* NRRL 502 in the larger analysis that included all 30 strains, whereas 283 were identified in the smaller analysis that included only 7 strains (Fig. 2). From our examination of all 30 strains, 1,991 orthogroups were unique to A. flavus strains, compared to over 3,000 orthogroups identified in the 7-strain analysis. No orthogroups were absent in A. flavus but present in all other species.

### Many predicted biosynthetic gene clusters and secondary metabolites were shared between species.

**FIG 3:**
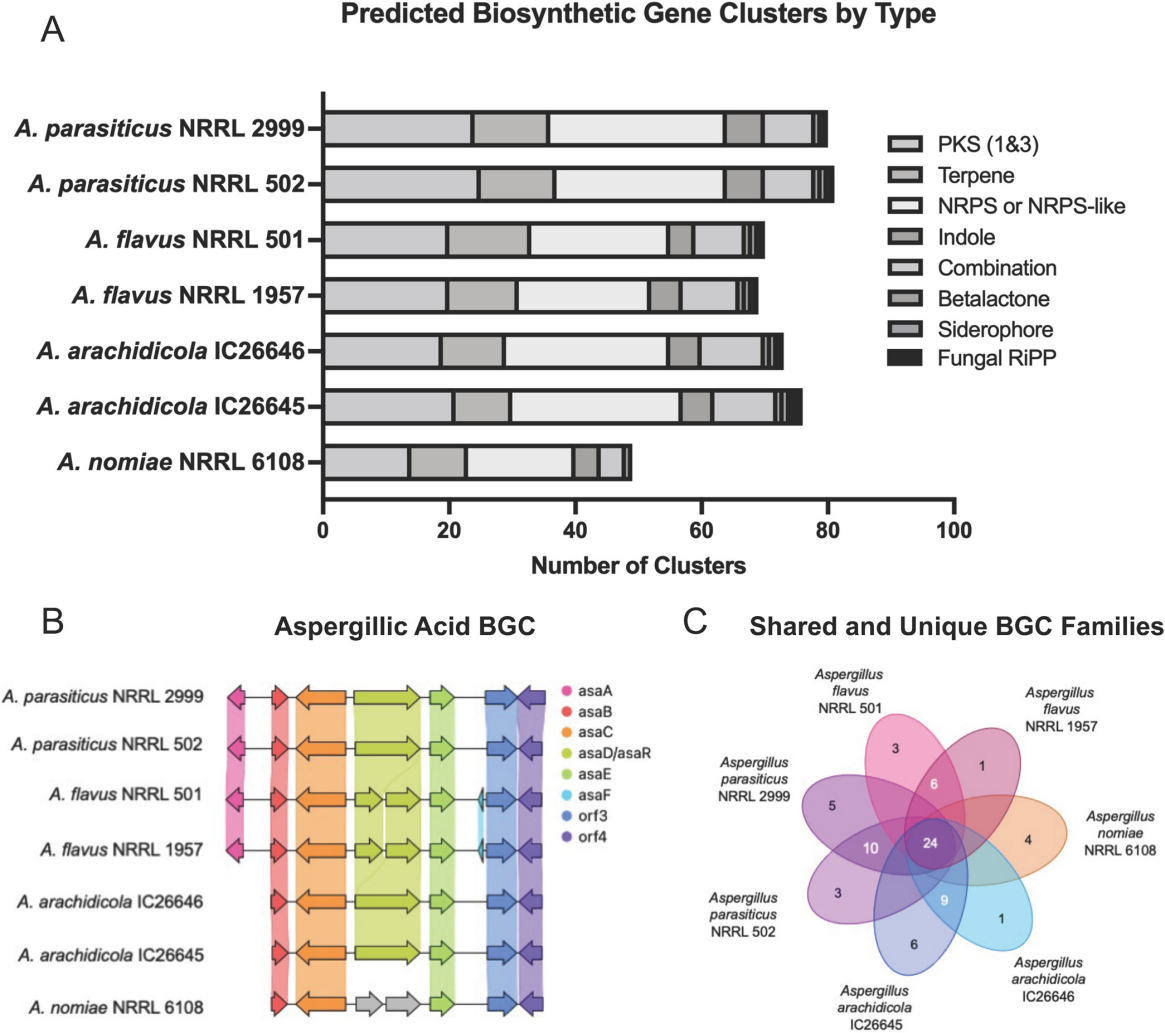
Strains of the same species did not differ substantially in their predicted biosynthetic gene clusters (BGCs). (A) Stacked bar plot of predicted BGCs. Each bar adds up to the total number of predicted BGCs, with the type of BGC indicated by color. (B) Synteny plot comparison of the aspergillic acid BGC from all seven strains. Arrows represent genes, and vertically shaded areas between arrows indicate sequence similarity. (C) Diagram of unique (singletons) and shared BGC families, calculated by BiGSCAPE.

Using the fungal version of antiSMASH, we predicted biosynthetic gene clusters (BGCs) from the seven strains. *A. nomiae* NRRL 6108 had the fewest predicted BGCs at 49, whereas *A. parasiticus* strains had the most predicted BGCs, as NRRL 2999 and NRRL 502 were predicted to contain 80 and 81 BGCs, respectively. Both *A. arachidicola* strains encoded more predicted BGCs (76 for IC26645 and 73 for IC26646) than either A. flavus strain, but fewer than either *A. parasiticus* strain. Our two A. flavus strains, NRRL 1957 and NRRL 502, encoded 70 and 71 BGCs, respectively (Fig. 3A). Of these BGCs, 44 (NRRL 1957) and 45 (NRRL 502) were not linked to any known metabolites.

A total of 118 BGC families were present in the seven strains, with 24 families shared by all strains and 7 families unique to the A. flavus strains (Fig. 3C). Eight additional BGC families were identified in all strains but *A. nomiae* (see Table S1 in our supplementary information [https://doi.org/10.6084/m9.figshare.20256336]). Our A. flavus strains shared 58 BGC families (see Table S1).

### Genetic determinants of virulence were present in all species.

**FIG 4:**
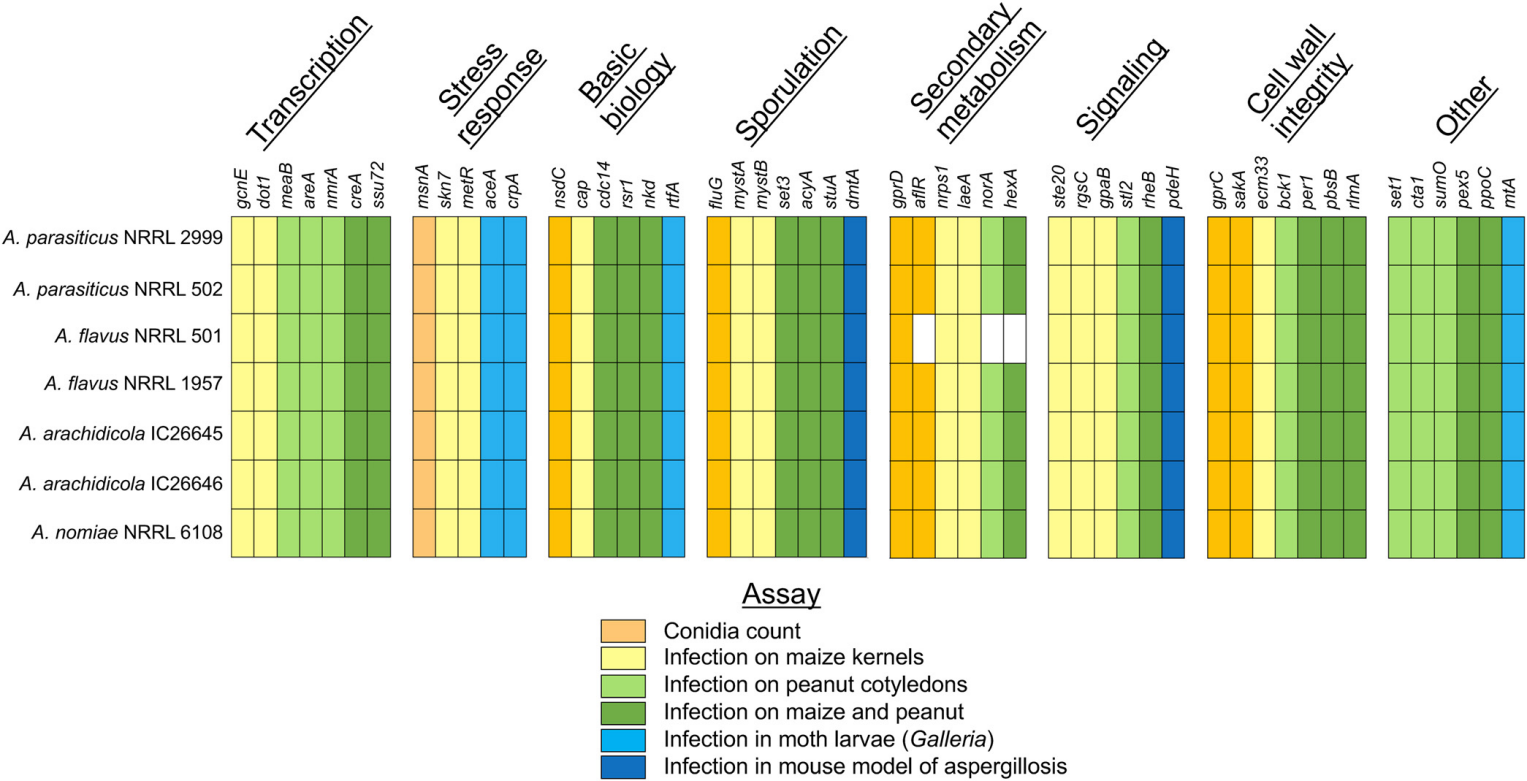
Genetic determinants of virulence were identified in all strains; data shown here summarize presence or absence of 50 Aspergillus flavus genetic determinants of virulence. All genes were identified in all strains except *aflR*, *norA*, and *hexA*, which were absent in A. flavus NRRL 501. Colors represent the A. flavus experimental virulence assay for each gene (from published literature). Genes which were studied using an animal model are in shown in light or dark blue. Additional information is provided in Table S2 of our supplementary information on the FigShare website.

Through a review of the literature, we identified 50 genes related to virulence or sporulation in A. flavus (29, 31–72). The majority of the genetic determinants of virulence have been studied only in the context of plant pathology, with seed infection and asexual spore (conidia) count assays, although six genes have been tested in animal models of fungal disease (Fig. 4). Several studies implicated aflatoxin biosynthesis in A. flavus plant pathogenesis, linking virulence and aflatoxin production. With the exception of genes involved in the aflatoxin biosynthetic gene cluster, which are absent in A. flavus NRRL 501, all 50 genetic determinants of virulence were identified in all seven strains (Fig. 4). Of the genes identified, 12 were orthologous to genetic determinants of virulence for A. fumigatus (see Table S2). An additional stress response protein, *sfgA*, which plays an important role in rendering A. flavus more stable to the external environment (73), was also found in all seven strains.

### Strains had heterogeneous secondary metabolite profiles.

**FIG 5:**
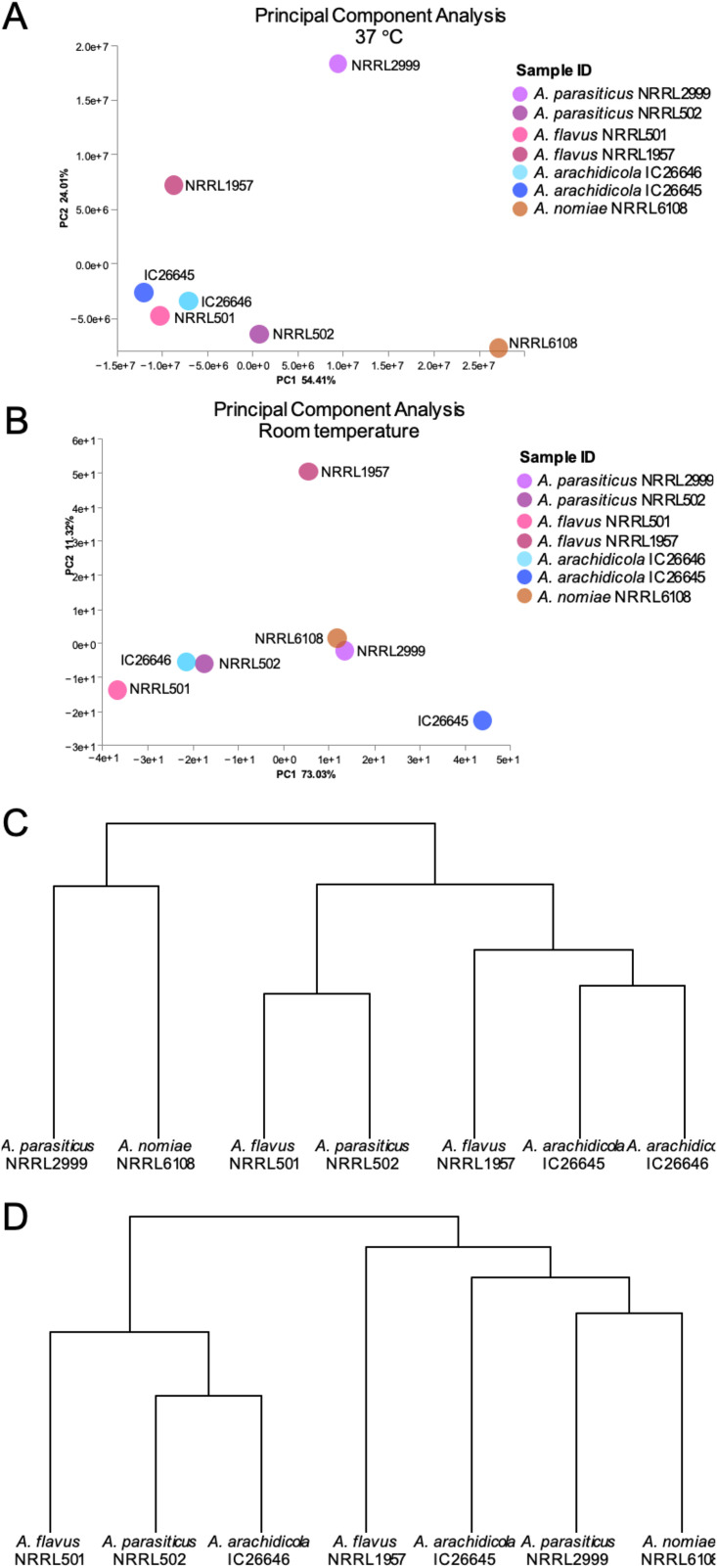
The metabolic profiles of Aspergillus flavus strains were more similar at 37°C than room temperature. The metabolomic profiles of A. flavus NRRL 501 and NRRL 1957 were almost identical at 37°C, showing very similar metabolites in the UPLC-MS analysis; most of the metabolites identified were fatty acids and ergosterol derivatives. In contrast, the profiles were significantly different at room temperature. (A) Principal-component analysis for all strains at 37°C. Circles represent both presence of metabolites and relative abundance. (B) Principal-component analysis for all strains at room temperature. Circles represent both presence of metabolites and relative abundance. (C) Hierarchical clustering of strains based on metabolite profiles at 37°C. (D) Hierarchical clustering of strains based on metabolite profiles at room temperature.

Evaluation of the secondary metabolite profiles of the seven fungi at both room temperature and 37°C, with or without the inclusion of saline, led to several key observations. First, the inclusion or absence of saline had little to no effect on the secondary metabolite profiles, as major differences were not observed between strains grown under the same conditions (i.e., room temperature or 37°C) both with and without physiologic saline (see Fig. S2 and S3 in our supplementary information [https://doi.org/10.6084/m9.figshare.20256336]). Therefore, our analysis of secondary metabolites focused on the impact of temperature. Each strain produced a unique set of metabolites, particularly at room temperature, and this held true even for strains of the same species (Table 3). A principal-component analysis (PCA) revealed that *A. parasiticus* strains NRRL 2999 and NRRL 502 were most differentiated along principal component two at 37°C, explaining ~24% of the variation (Fig. 5A). Conversely, strains of *A. arachidicola* were more similar to each other at 37°C than at room temperature, as were strains of A. flavus (Fig. 5).

**TABLE 3:**
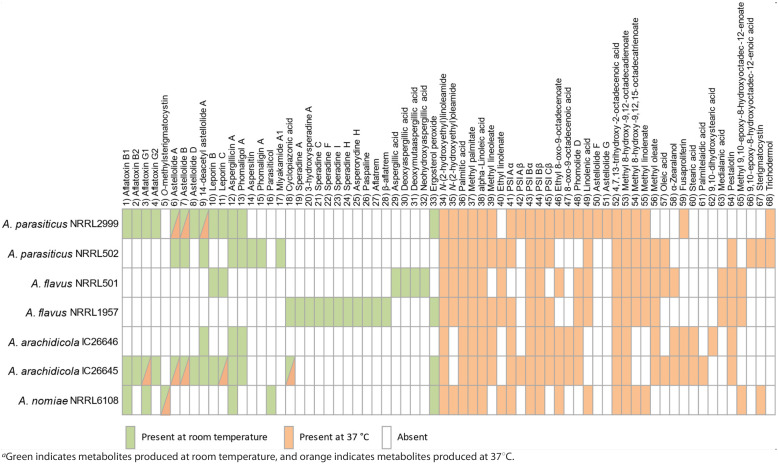
Variation in the presence or absence of secondary metabolites in the seven strains from four Aspergillus species^*a*^

In general, the cultures grown at room temperature contained a high proportion of mycotoxins (i.e., compounds **1** to **32**) or ergosterol derivatives (**33**), whereas the cultures grown at 37°C had a much higher concentration of metabolites derived from fatty acids (i.e., compounds **34** to **68**) (further details for compounds **1** to **68** have been submitted to the Natural Products Magnetic Resonance Database [https://np-mrd.org/]). Interestingly, the two strains of A. flavus (i.e., NRRL 501 and NRRL 1957) did not share any compounds in their secondary metabolite profiles when grown at room temperature. Moreover, other than leporin B and C (compounds **10** and **11**, respectively) and cyclopiazonic acid (**18**), the secondary metabolite profiles of the two strains of A. flavus did not overlap with the secondary metabolite profiles of any of the other section *Flavi* species when grown at room temperature. A. flavus NRRL 501 largely biosynthesized aspergillic acid (**29**) and a variety of related analogs, confirming previous reports (74), whereas A. flavus strain NRRL 1957 biosynthesized cyclopiazonic acid (**18**) and a range of analogs, such as speradines A (**19**), C (**21**), F (**22**), I (**23**), and H (**24**) and asperorydine H (**25**). Additionally, this strain biosynthesized indol-terpenoids, such as paspaline (**26**) and aflatrem (**27**).

*A. parasiticus* NRRL 2999 and *A. arachidicol*a IC26645 had very similar secondary metabolite profiles and were prolific producers of aflatoxins B1 (**1**), B2 (**2**), G1 (**3**), and G2 (**4**), astellolids A (**6**), B (**7**), and D (**8**), and leporins B (**10**) and C (**11**), along with some minor metabolites, such as aspergillicin A (**12**) and phomaligol A (**13**). In contrast, *A. parasiticus* NRRL 502 and *A. arachidicola* IC26646 did not biosynthesize aflatoxins or leporins. However, *A. parasiticus* NRRL 502 produced astellollids A (**6**) and B (**7**) and polyketides like phomaligol A (**13**), phomagilin A (**14**), and aspersitin (**15**), whereas the latter (i.e., *A. arachidicola* IC26646) only produced the 14-deacetyl derivative of astellolide A (**9**) and traces of aspergillicin A (**12**) and phomaligol A (**13**). Furthermore, we observed that *A. nomiae* NRRL6108 biosynthesized a smaller suite of mycotoxins, which was consistent with the smaller number of predicted BGCs in its genome; however, it uniquely produced *O*-methylsterigmatocystin (**5**) and parasiticol (**16**), two metabolites closely related to aflatoxins and likely derived from similar BGCs.

The isolated metabolites at 37°C were largely composed of fatty acids (mostly C_18_ and C_16_ derivatives), particularly the sporogenic PSI (precocious sexual inducer) factors A, B and C (i.e., **41** to **45**). These compounds were found in all the strains grown at 37°C. A few of the mycotoxins were also observed at elevated temperatures, specifically astellolids A (**6**) and B (**7**) in *A. parasiticus* NRRL 2999 and *A. arachidicola* IC26645 and *O*-methylsterigmatocystin (**5**) in *A. nomiae* NRRL 6108. In total, the results showed a surprising amount of heterogeneity of the secondary metabolomic profiles both between species and even between strains of the same species. Structures for all identified metabolites are available in our supplementary information on FigShare (Table S3) and were identified through extensive comparison to available structures from the literature (35, 75–99).

### A. flavus strains responded differently to cell wall stress and antifungal drug resistance but similarly to hypoxia and iron starvation.

**FIG 6:**
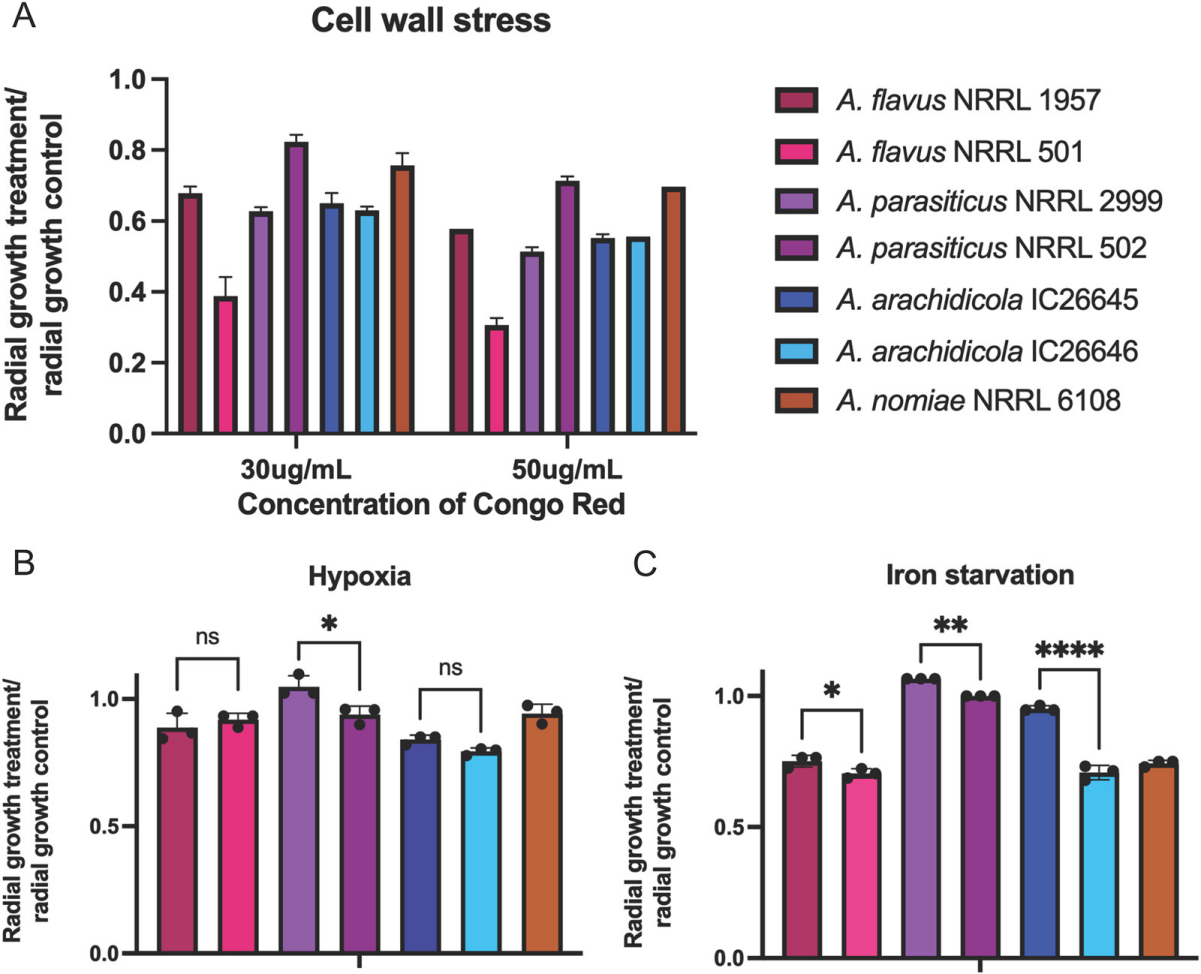
Iron starvation and cell wall stress impacted growth of A. flavus strains differently, but hypoxic conditions impacted A. flavus strains similarly. For each of the seven Aspergillus strains, radial growth is expressed as a ratio of colony radial diameter (in centimeters) of growth under the stress condition divided by the colony radial diameter in the control (solid minimal medium). Not all significant comparisons are shown. (A) Hypoxic stress was induced by incubating plates in 1% O_2_ and 5% CO_2_. Statistical significance of growth differences among species was primarily driven by growth of *A. parasiticus* NRRL 2999, which was significantly less impacted by hypoxia than other strains. (B) Iron starvation was induced through growth on iron-depleted substrate in the presence of gallium. All species with multiple strains exhibited strain heterogeneity, and *A. parasiticus* grew significantly better under iron starvation conditions than other species. Other species comparisons between species were nonsignificant. (C) Cell wall perturbation was induced by adding Congo red to the medium. A. flavus NRRL 501 was most impacted by Congo red at both concentrations, and *A. parasiticus* NRRL 502 was impacted the least. At both concentrations, strains of A. flavus had significantly different responses to cell wall stress (*P* < 0.0001). Cell wall stress also impacted *A. parasiticus* strains differently (*P* < 0.0001). Strains of *A. arachidicola* did not have significant growth differences. ns, not significant; *, *P* ≤ 0.05; **, *P* < 0.005; ***, *P* < 0.0005 (ANOVA).

Hypoxia impacted growth of all strains negatively, but *A. parasiticus* NRRL 2999 was significantly less impacted than all other strains (*P* = 0.0346). Growth of *A. arachidicola* IC26646 was most impacted by the hypoxic environment, although it did not significantly differ from *A. arachidicola* IC26645 or A. flavus NRRL 1957. Responses of *A. parasiticus* strains to hypoxia were significantly different (*P* = 0.027), whereas observed differences between strains of A. flavus and between *A. arachidicola* strains were not (Fig. 6A).

Under iron starvation conditions (growth in iron-depleted medium), *A. parasiticus* NRRL 2999 was least impacted and grew slightly better than it did in iron-supplemented medium. Growth of A. flavus NRRL 501 was most impacted by the lack of iron (Fig. 6B). A. flavus strains differed significantly from *A. parasiticus* strains under iron starvation conditions (*P* < 0.0001, one-way analysis of variance [ANOVA]) and from one another (*P* = 0.0398). Growth differences of the two *A. parasiticus* strains under iron starvation conditions were also statistically significant (*P* = 0.0030), as were dissimilarities in growth rates of the two *A. arachidicola* strains (*P* < 0.0001). A. flavus NRRL 1957 was more sensitive to oxidative stress than the other strains (*P* = 0.0003).

Strains of A. flavus had varied responses to cell wall stress, with a stronger impact of Congo red on the growth of NRRL 501 than that of NRRL 1957 (*P* < 0.0001, two-way ANOVA). A. flavus NRRL 501 was significantly more sensitive to cell wall stress than any other strain (*P* < 0.0001). *A. parasiticus* strains also exhibited different growth rates in the presence of Congo red, with NRRL 2999 growing less than NRRL 502 (*P* < 0.0001). Growth of the two *A. arachidicola* strains was not significantly different between the two or from A. flavus NRRL 1957 for either concentration of Congo red (Fig. 6C).

Aspergillus parasiticus NRRL2999 had the lowest MIC of amphotericin B (0.5 μg/mL), with all other strains requiring a higher dose to inhibit growth (1 μg/mL). Both *A. arachidicola* strains and *A. nomiae* NRRL 6108 had a higher MIC of voriconazole, with *A. parasiticus* and A. flavus strains being more susceptible (Table 4).

**TABLE 4:** MICs of the antifungal drugs amphotericin B and voriconazole for the seven strains of four Aspergillus species

Strain	MIC (μg/mL)	
	Amphotericin B	Voriconazole
Aspergillus *arachidicola* IC26645	1.0	1.0
Aspergillus *arachidicola* IC26646	1.0	1.0
Aspergillus flavus NRRL1957	1.0	0.5
Aspergillus flavus NRRL 501	1.0	0.5
Aspergillus *nomiae* NRRL 6108	1.0	1.0
Aspergillus parasiticus NRRL 502	1.0	0.5
Aspergillus parasiticus NRRL 2999	0.5	0.5

### A. flavus is not significantly more virulent than related, nonpathogenic species in an invertebrate model of fungal disease.

Using an invertebrate model of fungal disease, we evaluated virulence for all strains. A. fumigatus virulence assays typically use concentrations of 1 × 10^6^ conidia (asexual spores) to inoculate Galleria mellonella larvae (41, 42). When we inoculated larvae with section *Flavi* species at 1 × 10^6^, all animals died within 2 days. Previous studies provided evidence that A. flavus kills G. mellonella larvae faster than A. fumigatus and therefore requires a lower inoculum (100). At a lower concentration of inoculum, 1 × 10^4^ conidia (spores), the larvae survived longer and differences among strains were apparent.

**FIG 7:**
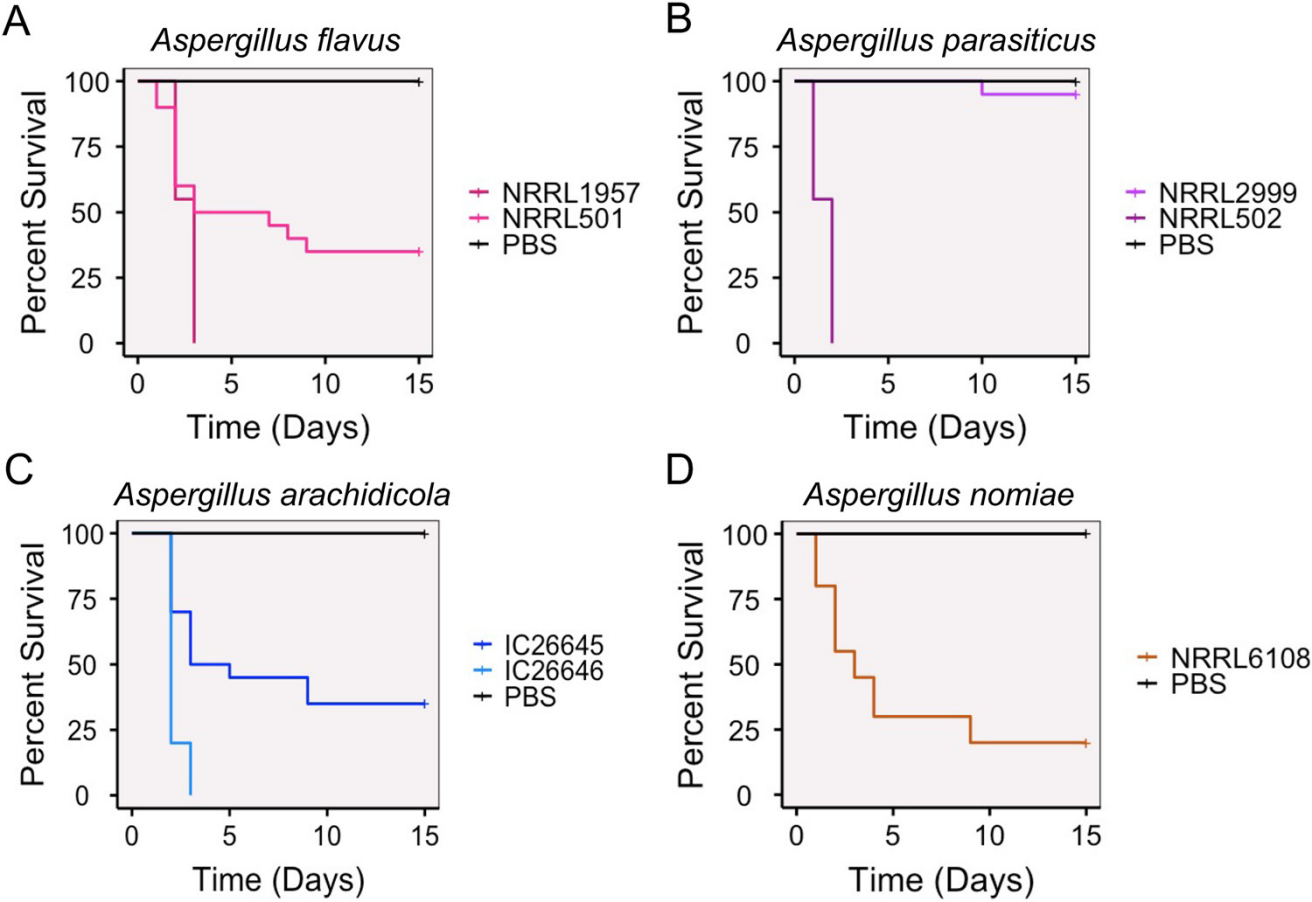
Aspergillus flavus was not significantly more virulent than related nonpathogenic species in an invertebrate model of fungal disease. Cumulative survival of Galleria mellonella larvae inoculated with 1 × 10^4^ asexual spores (conidia) of an Aspergillus strain or a PBS control. (A) Survival for larvae inoculated with either A. flavus NRRL 1957 or NRRL 501. All pairwise comparisons between the two strains and the control group were statistically significant. (B) Survival for larvae inoculated with either *A. parasiticus* NRRL 2999 or NRRL 502. *A. parasiticus* NRRL 2999 was not statistically different from the control group, but NRRL 502 was statistically different from both the control and NRRL 2999. (C) Survival for larvae inoculated with either *A. arachidicola* IC26645 or IC26646. All pairwise comparisons between the two strains and the control group were statistically significant. (D) Survival for larvae inoculated with *A. nomiae* NRRL 6108. Survival of NRRL 6108 was statistically different from the control survival.

We found that strains of the same species varied widely in their virulence profiles and that strains of the pathogen A. flavus were not more virulent than strains of the nonpathogenic species (Fig. 7). Using a 1 × 10^4^ concentration of spores, *A. parasiticus* NRRL 2999 killed the fewest animals and was not statistically different from the phosphate-buffered saline (PBS) control injections. In contrast, *A. arachidicola* IC26646, A. flavus NRRL 1957, and *A. parasiticus* 501 killed all inoculated moth larvae by day 3. Of note, the three most virulent strains were all from different species, as were the three least virulent strains; strains of the same species killed larvae at significantly different rates (Fig. 7).

### Section *Flavi* strains infected eyes as well as A. fumigatus *in a murine model of fungal keratitis*.

**FIG 8:**
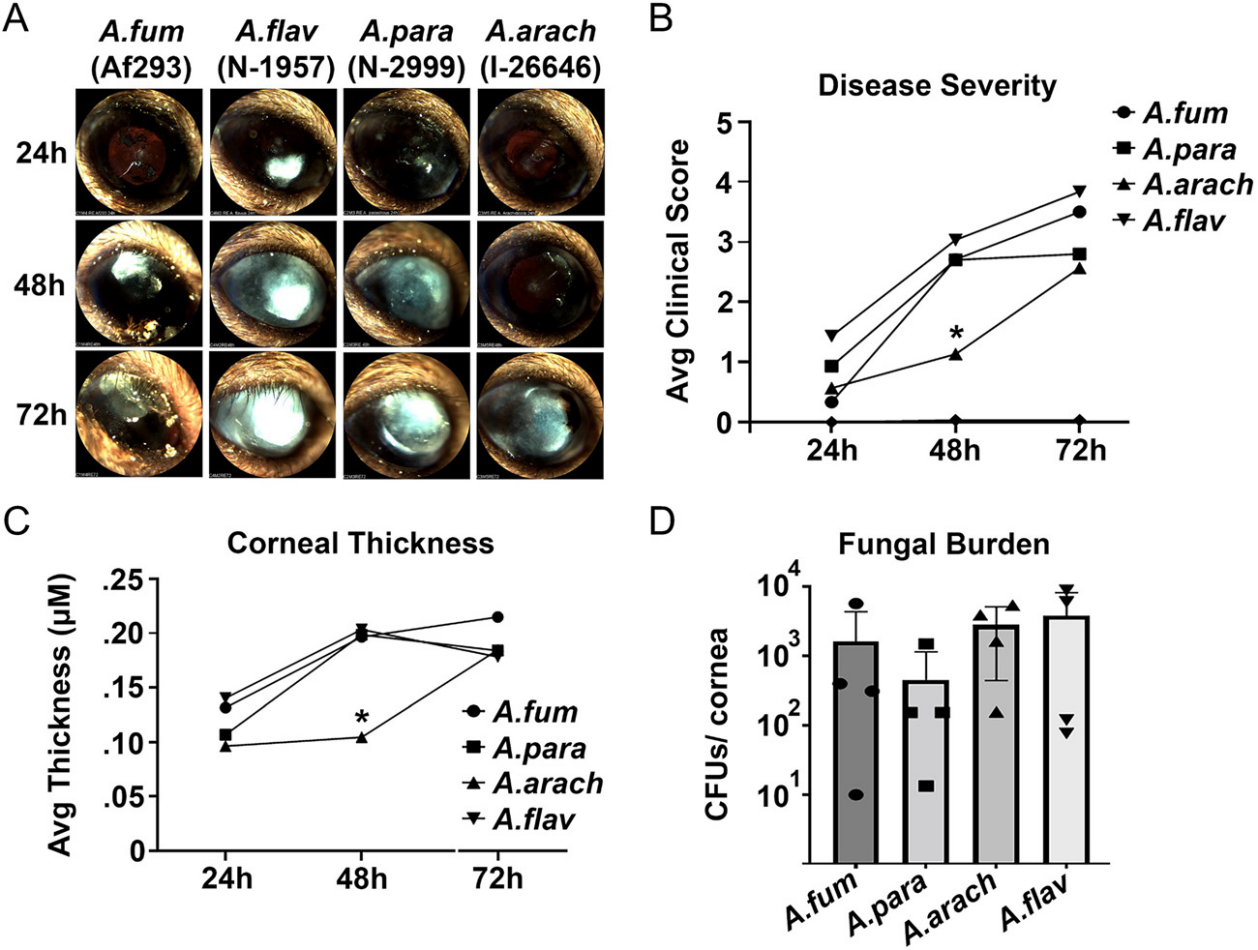
Section *Flavi* species infected eyes as well as A. fumigatus did in a murine model. (A) Slit-lamp images of a representative animal for each infection group. (B) Clinical score analysis of all slit-lamp images revealed reduced disease severity in the *A. arachidicola* group at 48 h postinfection (*n* = 5/group). (C) Corneal thickness measured by optical coherence tomography similarly revealed reduced structural alteration in the *A. arachidicola* group at 48 h (*n* = 5). (D) CFU analysis on resected corneas revealed indistinguishable fungal burden at 72 h postinfection between infection groups. *A.arach*, *A. arachidicola* IC26646; *A.flav*, A. flavus NRRL 1957; *A.fum*, A. fumigatus Af293; *A.para*, *A. parasiticus* NRRL 2999.

Finally, we used a mouse model of keratitis to compare one strain from each of the four different Aspergillus species at 24, 48, and 72 h postinfection (Fig. 8A). We included A. fumigatus Af293 along with the three section *Flavi* species (*A. arachidicola* IC26646, A. flavus NRRL 1957, and *A. parasiticus* NRRL 2999) to benchmark the virulence of section *Flavi* species against the reference strain for section *Fumigati*. We observed significantly lower disease severity (Fig. 8B) and corneal thickness (Fig. 8C) in *A. arachidicola* IC26646 infections compared to the those with other species at 48 h postinfection, but this difference was not significant at 72 h. Fungal burden was not significantly different between the four species (Fig. 8D).

## DISCUSSION

With the goal of studying the evolution of pathogenicity in section *Flavi*, we examined the genomes, chemotypes, and phenotypes of four closely related species: the major pathogen A. flavus and three related nonpathogenic species, *A. arachidicola*, *A. parasiticus*, and *A. nomiae.* We observed similarities and differences between A. flavus and the three nonpathogenic relatives, including shared gene content, variable production of secondary metabolites, and virulence in two animal disease models.

Genomic content was highly similar between strains of the same species, with few strain-specific protein families identified. Predicted BGCs were similar between strains of the same species, with 21 shared by all strains. Georgianna et al. (101) previously predicted 55 BGCs from A. flavus NRRL 3357. Of these, 14 BGCs have been linked to a specific metabolite. Additional clusters producing kojic acid, aflavinines, apseripin-2a, and ustiloxins were later identified. Recently, Drott et al. (102) studied 94 different A. flavus strains and identified 92 unique BGCs. Our A. flavus strains were predicted to contain 70 to 71 BGCs, well within the expected range, although over half of the predicted BGCs in each strain have not been linked to known metabolites. Our results of the number and type of predicted BGCs per strain are in line with results from Kjærbølling et al. (31), who also found that *A. parasiticus* had more predicted biosynthetic backbone genes than *A. arachidicola*, A. flavus, or *A. nomiae*. However, the *A. nomiae* strain studied by Kjærbølling et al. (NRRL 13137) was predicted to encode more backbone genes than our strain (NRRL 6108), further highlighting strain-level differences within species.

Among the species with multiple strains included, the two *A. parasiticus* strains shared the fewest species-specific protein families (1,230), whereas pairs of strains from *A. arachidicola* and A. flavus shared over 3,000 species-specific protein families between strains, around 20% of the total number of protein families for each species. Our results within section *Flavi* contrast with observations within section *Fumigati*, which show a lower proportion of species-specific genes for A. fumigatus than in related, nonpathogenic species (103). Specifically, when strains of A. fumigatus were compared to other section *Fumigati* species, only 72 families were identified as unique to A. fumigatus (103); in contrast, we observed over 1,000 unique families in A. flavus. Although section *Flavi* species are known to have larger genomes and encode more predicted proteins than A. fumigatus (31), this does not explain the huge difference in number of species-specific protein families between A. fumigatus and A. flavus and further emphasizes that the two sections are quite distinct in their genomic compositions.

Resistance to antifungal drugs was also similar between strains of the same species and within previously reported ranges for A. flavus (104). Our A. flavus and *A. parasiticus* strains were more susceptible to amphotericin B than clinical strains of the same species from Brazil (105), and strains from all four species had similar susceptibility to voriconazole as clinical strains from Brazil (106, 107). As expected, our *Flavi* strains were generally more resistant to amphotericin B than to voriconazole.

Several traits that we examined revealed heterogeneity between strains of the same species, indicating diversity within each species as well as among species. Our two A. flavus strains, NRRL 501 and NRRL 1957, for example, did not produce any of the same secondary metabolites when grown at room temperature, and only a handful of compounds produced by the two strains at room temperatures overlapped with those produced by strains of other species. We found that our *A. parasiticus* strains produced aflatoxin at higher levels than other species, including noted aflatoxin producer *A. parasiticus* NRRL 2999, although one strain of A. flavus, NRRL 1957, had previously been characterized as negative for aflatoxin (107). At the temperature of the human body, which better models infection-relevant conditions, several compounds were produced by all seven strains. These included fatty acids such as palmitic acid, which has been implicated in inflammation (108), and precocious sexual inducer factors. Interestingly, our two A. flavus strains had highly similar chemical profiles at 37°C.

Strains of the same species also responded differently to environmental stressors in growth assays, including cell wall stress and iron starvation, which may affect a strain’s fitness within a human host. For example, the ability to tolerate cell wall stress (induced by Congo red) correlated with higher virulence in an invertebrate model, as observed in A. flavus NRRL 1957 and *A. parasiticus* NRRL 502. Genetic determinants of virulence in A. flavus, including those involved in cell wall integrity, stress response, and transcription, were found in all strains. Virulence-associated genes involved in secondary metabolism were missing in A. flavus NRRL 501 as a result of the aflatoxin biosynthetic gene cluster absence in the strain. Several genetic determinants of virulence within A. flavus influence aflatoxin production as well as conidiation and fungal development (43, 47, 52, 56, 58), with multiple genes within the pathway itself directly implicated in virulence (69, 72), indicating aflatoxin production is intertwined with basic biological functions of the fungus. Therefore, aflatoxin production may also impact pathogenicity in animals, although few studies include animal models and current study of virulence within A. flavus is dominated by seed inoculation assays and spore counts that are of relevance to its role in plant disease. However, knockout mutants of some genes have been shown to decrease virulence in both plant and animal models of infection (42), demonstrating parallels between the plant pathogenesis and animal pathogenesis, a phenomenon also seen in other opportunistic fungal pathogens (109).

Surprisingly, in Galleria mellonella, neither A. flavus strain was the most virulent, and all species were able to infect and kill at least some larvae, although *A. parasiticus* NRRL 2999 killed only 10% of larvae. As observed previously, the concentration of asexual spores required to kill all larvae within 24 h was orders of magnitude lower for infection with section *Flavi* species compared to section *Fumigati* species (110), suggesting higher virulence in the section *Flavi* species. In the most virulent section *Flavi* strains (*A. parasiticus* NRRL502, A. flavus NRRL1957, and *A. arachidicola* IC26646), death of all larvae occurred within 72 h. A previous study using a lower concentration inoculum (1 × 10^3^ spores) saw 100% mortality of larvae in the first 48 h for *Galleria* infected with A. flavus (110). However, in another study of A. flavus strains, also with a concentration of 1 × 10^3^ spores, 10% or more of the larvae were alive after 72 h for all strains (111). Few studies of non-A. flavus section *Flavi* species have included virulence assays in *Galleria*, presumably due to their status as nonpathogenic, but this prevents direct comparison between our results and previous studies for species other than A. flavus. As a known entomopathogen (112), virulence by A. flavus in an invertebrate model may be correlated with insecticidal traits rather than (or in addition to) human pathogenicity.

To examine virulence in a mammalian model of fungal keratitis, we infected eyes of mice with one strain each of A. flavus, *A. arachidicola*, and *A. parasiticus*, along with one A. fumigatus strain. We observed faster colonization of mice by A. flavus than A. fumigatus at 24 h, although fungal burden and disease severity were consistent for all species by 72 h. *A. arachidicola* IC26646 did not infect as quickly, with significantly lower disease severity observed at 48 h postinfection, but the level caught up to the others by 72 h postinfection. Mouse models of disease are a useful tool for understanding disease progression and characterizing differences among strains, but previous studies have induced systemic infections leading to mouse death (113). Systemic infection studies have shown A. flavus to be more virulent than A. fumigatus (24, 113), despite higher rates of aspergillosis caused by A. fumigatus. Interestingly, we observed no difference in disease severity at 72 h between the two species in our keratitis model. The similarity of secondary metabolite profiles for strains at 37°C, coupled with the ability of all species to establish infections in both the *Galleria* and murine models, leads us to believe that infection may be more highly dependent on the characteristics of the individual strain rather than species, as observed in other pathogens among Aspergillus (114). We intend to compare additional strains of both A. flavus and A. fumigatus, including patient-derived clinical strains, to examine differences in virulence and disease progression of A. flavus and A. fumigatus in our keratitis model. It remains an open question whether patient-derived clinical strains of A. flavus differ from the environmental strains available from culture collections, such as the two strains included in this study, and we hope to explore this dimension of pathogenicity in future studies.

In summary, by examining genomic, chemical, and phenotypic variation within and between closely related pathogenic and nonpathogenic Aspergillus section *Flavi* species, we showed that species previously considered nonpathogenic infect at the same rate as the pathogen A. flavus in both invertebrate and murine models of disease and, in the case of *A. parasiticus* NRRL 2999, showed that a single strain may be considered avirulent in one model and virulent in another. Therefore, we advocate for the use of multiple disease models for the future study of fungal disease caused by species within section *Flavi*. Our results indicate that strain-level differences may play a major role in infection. Additionally, we showed that predicted biosynthetic gene clusters and genetic determinants of virulence do not differ substantially between strains and therefore do not explain the differences in virulence, possibly implicating as-yet-uncharacterized variations in gene presence or absence between strains and species and/or transcriptomic or posttranslational regulatory mechanisms in the occurrence and prevalence of human disease caused by Aspergillus species, particularly A. flavus.

## MATERIALS AND METHODS

### Genome sequencing and comparisons.

**(i) Strains and growth conditions.** Seven strains from four Aspergillus species were obtained from the USDA Agricultural Research Service Culture Collection (A. flavus NRRL 501, Aspergillus *nomiae* NRRL 6108, and Aspergillus parasiticus NRRL 2999) or from the laboratory of Ignazio Carbone at North Carolina State University (Aspergillus flavus NRRL 1957, Aspergillus parasiticus NRRL 502, and Aspergillus *arachidicola* IC26645 and IC26646). All strains were maintained on potato dextrose agar (PDA; Difco) at room temperature (approximately 23°C).

**(ii) Genomic DNA extraction and sequencing.** For genomic DNA extraction to facilitate genome sequencing, the strains were first grown on PDA. Each strain was subsequently transferred to a 100-mm petri dish with an overlay of sterile autoclaved Hybond nylon membranes (84 mm) obtained from Amersham (GE Healthcare). The strains were then allowed to grow for 2 to 3 weeks on PDA medium overlaid with this membrane. After suitable growth was achieved, a sterile scalpel was used to harvest the fungal mycelia by scraping the surface of the nylon membrane without dislodging any agar from the petri plate. Using a sterile mortar and pestle, the mycelia were then ground to a fine powder with liquid nitrogen and transferred to a bashing bead tube with 750 μL of DNA lysis buffer (Zymo Quick-DNA fungal/bacterial miniprep kit). The powder in the bashing bead tube was further disrupted and homogenized in a Qiagen tissue lyser LT bead mill for 5 min. Genomic DNA was obtained using the standard protocol for the Zymo Quick-DNA fungal/bacterial miniprep kit.

Paired-end sequencing (2 × 150 bp) of the genomic DNA was performed at the Vanderbilt Technologies for Advanced Genomics (VANTAGE) facility using the NovaSeq 6000 platform (Illumina, Inc.) following the manufacturer’s protocols. Libraries were prepared using the Illumina TruSeq DNA PCR-free kit.

**(iii) Genome assembly, annotation, and assessment.** Adaptors were removed from the reads and filtered for quality using Trimmomatic v0.39 (115). Trimmed reads were assembled into draft genomes using SPAdes v3.12.0 (116) with the “–careful” flag. Scaffolds under 500 bp were removed from further analysis. Draft genomes were annotated using Liftoff v1.2.0 (117), a sequence-similarity method which requires a reference annotation. A. flavus strains and *A. nomiae* NRRL 6108 were annotated using strain NRRL 3357 (118); for *A. arachidicola* strains we used CBS 117612 (31); *A. parasiticus* strains were annotated from CBS 117618 (31). The *A. parasiticus* NRRL 2999 draft genome was compared to that of *A. parasiticus* SU-1 to confirm isolate identity using fastANI (119), which compares average nucleotide identity (ANI) between genomes. Genome and annotation completeness was evaluated using BUSCO v4.0.4 (120), against the eurotiales database of universal single-copy orthologs.

**(iv) Phylogenetics.** In addition to the 7 newly sequenced genomes, the genomes and predicted proteomes of 23 other Aspergillus strains were downloaded from NCBI (Table 5). In total, we used 29 strains from 18 species of section *Flavi* to build a species tree phylogeny of Aspergillus section *Flavi*, with Aspergillus niger CBS 513.88 (section *Nigri*) serving as an outgroup. Single-copy orthologous proteins in all 30 strains were identified using OrthoFinder v2.5.4 (121). For each ortholog, all 30 copies were aligned using Muscle v3.8.1551 (122), and the amino acid alignments were trimmed using trimAl v1.2 (123) with the gappyout option. Alignments shorter than 200 amino acids were removed from analysis, and the remaining alignments were concatenated into a single data matrix using the script catfasta2phyml.pl (https://github.com/nylander/catfasta2phyml). A maximum likelihood phylogeny was reconstructed using IQ-tree v1.6.1 (124) with the JTT+F+I+G4 model and 1,000 replicates for bootstrapping. The resulting consensus tree was viewed using iTOL (125).

**TABLE 5:** Genomes of 27 Aspergillus strains from 20 species, including 7 newly sequenced genomes^*a*^

Species	Strain	Reference	Environmental source
Aspergillus alliaceus	CBS 536.65^T^	Kjærbølling et al., 2020	Dead blister beetle, Washington, DC, USA
Aspergillus arachidicola	CBS 117610^T^	Kjærbølling et al., 2020	
Aspergillus *arachidicola*	CBS 117612	Kjærbølling et al., 2020	
Aspergillus *arachidicola*	IC26645 (IBT 27218)	This study	Unknown
Aspergillus *arachidicola*	IC26646 (IBT 27178; CBS 117615)	This study	*Arachis glabrata* leaf, Argentina
Aspergillus avenaceus	IBT 18842	Kjærbølling et al., 2020	Unknown
Aspergillus bertholletius	IBT 29228	Kjærbølling et al., 2020	
Aspergillus luteovirescens	NRRL 26010^T^ (CBS 117187)	Kjærbølling et al., 2020	Frass in a silkworm rearing house, Japan
Aspergillus caelatus	CBS 763.97^T^	Kjærbølling et al., 2020	Peanut field, GA, USA
Aspergillus coremiiformis	CBS 553.77^T^	Kjærbølling et al., 2020	Unknown
Aspergillus flavus	CBS 121.62	Kjærbølling et al., 2020	Unknown
Aspergillus flavus	NRRL 501	This study	Unknown
Aspergillus flavus	NRRL 1957^T^ (CBS 100927)	This study	Cellophane diaphragm of optical mask, South Pacific
Aspergillus flavus	NRRL 3357	Nierman et al., 2005	Moldy peanuts
Aspergillus leporis	CBS 151.66^T^	Kjærbølling et al., 2020	Dung of *Lepus townsendii*, WY, USA
Aspergillus minisclerotigenes	CBS 117635^T^	Kjærbølling et al., 2020	Arachis hypogaea seed, Argentina
Aspergillus niger (outgroup – section *Nigri*)	CBS 513.88	Pel et al., 2007	
Aspergillus nomiae	NRRL 13137	Moore et al., 2015	Wheat, IL, USA
Aspergillus *nomiae*	NRRL 6108	This study	Moldy wheat
Aspergillus novoparasiticus	CBS 126849	Kjærbølling et al., 2020	
Aspergillus parasiticus	NRRL 502^T^ (CBS 100926)	This study	Mealybug on sugar cane, HI, USA
Aspergillus parasiticus	NRRL 2999	This study	Peanuts, Uganda
Aspergillus parasiticus	SU-1	Linz et al., 2014	Peanuts, Uganda
Aspergillus parasiticus	CBS 117618	Kjærbølling et al., 2020	
Aspergillus pseudocaelatus	CBS 117616	Kjærbølling et al., 2020	Arachis hypogaea, Nigeria
Aspergillus pseudonomiae	CBS 119388 (NRRL 3353)	Kjærbølling et al., 2020	Diseased alkali bees, USA
Aspergillus pseudotamarii	CBS 117625	Kjærbølling et al., 2020	
Aspergillus sergii	CBS 130017	Kjærbølling et al., 2020	
Aspergillus tamarii	CBS 117626	Kjærbølling et al., 2020	
Aspergillus transmontanensis	CBS 130015	Kjærbølling et al., 2020	

a CBS, CBS-KNAW Fungal Biodiversity Centre, Utrecht, the Netherlands; IBT, IBT Culture Collection of Fungi, Lyngby, Denmark; NRRL, USDA-ARS Culture Collection, Peoria, IL, USA.

**(v) Identification of orthologous and unique protein families and biosynthetic gene clusters.** Using the seven newly sequenced strains (A. flavus NRRL 501 and NRRL 1957, *A. parasiticus* NRRL 502 and NRRL 2999, *A. nomiae* NRRL 6108, and *A. arachidicola* IC26645 and IC26646), we compared each predicted proteome in a pairwise manner and in combination to identify unique and orthologous proteins for all combinations using OrthoVenn2 (126). Within the unique or orthologous protein groups, enriched GO categories were identified from UniProt annotations (127), and probability testing was based on hypergeometric distribution (126). The R package UpSetR was used to visualize the number of shared protein families in each grouping as an upset plot. BGCs were predicted using fungiSMASH v6.0 (128). BGCs from different strains were compared using BiGSCAPE-CORASON (129) with a cutoff set to 0.70, and BGCs shared between all strains and strains of the same species were identified. Synteny plots of BGCs of interest were visualized using Clinker (130).

### Secondary metabolite isolation and structural elucidation.

The profile of secondary metabolites for the seven Aspergillus section *Flavi* strains were investigated using well-established procedures (26). Each strain was grown at both 23°C (i.e., room temperature) and 37°C on oatmeal (Quaker Oats old-fashioned breakfast oats). Since the lacrimal fluid was composed of physiologic saline, the oatmeal cultures were prepared both with and without 6 mg/mL saline to assess if saline impacted the profile of secondary metabolites.

Solid-state fermentations (*n* = 6) were carried out in broad-mouthed 250-mL Erlenmeyer flasks. To start oatmeal cultures, an agar plug from the leading edge of a PDA petri dish culture was transferred to a sterile tube with 10 mL of liquid yeast extract-soy-dextrose (YESD; 20 g soy peptone, 20 g dextrose, 5 g yeast extract, in 1 liter distilled H_2_O) and grown for 7 days on an orbital shaker (100 rpm) at room temperature (~23°C), and then used to inoculate the oatmeal solid fermentation medium. Oatmeal cereal medium (Quaker Oats) was prepared by adding 10 g oatmeal to a 250-mL Erlenmeyer flask with either 15 to 17 mL of deionized H_2_O (DI-H_2_O) or 15 to 17 mL of a 6-mg/mL saline solution (i.e., 6 g of Instant Ocean in 1,000 mL of DI-H_2_O) and then autoclaving at 121°C for 30 min. For each of the seven strains, six fermentation flasks were incubated at room temperature with saline and six without saline; similarly, six flasks were incubated at 37°C with saline and six without saline. Therefore, for each strain, 24 Erlenmeyer flasks were grown in total. Prior to analysis of the secondary metabolite profiles, the cultures were incubated statically at room temperature for 14 days or 37°C for 7 days.

**(i) Extraction of secondary metabolites.** For each condition, each of the six culture flasks was extracted individually and treated as a biological replicate. Each individual flask was extracted by adding 60 mL of CHCl_3_-methanol (MeOH; 1:1), chopping with a spatula, and shaking overnight (~16 h) at 100 rpm at room temperature. The cultures were then filtered *in vacuo*, and 90 mL of CHCl_3_ and 150 mL of DI-H_2_O were added to each of the filtrates. The mixtures were then transferred to a separatory funnel and shaken vigorously. The organic layer (i.e., bottom layer) was drawn off and evaporated to dryness *in vacuo*. The dried organic layer was reconstituted in a 100-mL mixture of CH_3_CN-MeOH (1:1) and 100 mL of hexanes, transferred to a separatory funnel, and shaken vigorously. The defatted organic layers (i.e., CH_3_CN-MeOH layers) were evaporated to dryness.

All the defatted organic layers were analyzed individually by ultraperformance liquid chromatography–high-resolution mass spectrometry (UPLC-HRMS), utilizing a Thermo LTQ Orbitrap XL mass spectrometer equipped with an electrospray ionization source. A Waters Acquity UPLC was utilized with a BEH C_18_ column (1.7 μm; 50 mm × 2.1 mm) set to a temperature of 40°C and a flow rate of 0.3 mL/min. The mobile phase consisted of a linear gradient of CH_3_CN−H_2_O (both acidified with 0.1% formic acid), starting at 15% CH_3_CN and increasing linearly to 100% CH_3_CN over 8 min, with a 1.5-min hold before returning to the starting conditions.

**(ii) Metabolomic analysis.** PCA and hierarchical clustering were performed on the UPLC-HRMS data. Untargeted UPLC-HRMS data sets for each sample were individually aligned, filtered, and analyzed using Mzmine 2.53 software (https://sourceforge.net/projects/mzmine/). Peak list filtering and retention time alignment algorithms were used to refine peak detection, and the join algorithm integrated all sample profiles into a data matrix using the following parameters: mass detection (MS1 level, centroid positive mode); ADAP chromatogram builder (group intensity threshold, 20,000; minimum highest intensity, 60,000; *m/z* tolerance, 0.003); chromatogram deconvolution (wavelets (ADAP) algorithm). For join aligner and gap filling the following parameters were used: retention time tolerance, 0.05 min; *m/z* tolerance, 0.0015 *m/z*. The resulting data matrix was exported to Excel (Microsoft) for analysis as a set of *m/z*-retention time (RT) pairs with individual peak areas. Samples that did not possess detectable quantities of a given marker ion were assigned a peak area of zero to maintain the same number of variables for all sample sets. Ions that did not elute between 1 and 10 min and/or had an *m/z* ratio of <200 or >900 Da were removed from analysis. Relative standard deviation was used to understand the quantity of variance between the injections, which may have differed slightly based on instrument variance. A cutoff of 1.0 was used at any given *m/z*-RT pair across the biological replicate injections, and if the variance was greater than the cutoff, it was assigned a peak area of zero. PCA and hierarchical clustering were conducted with Python. The PCA score plots were generated using the averaged data of the six individual biological replicates.

**(iii) Isolation and identification of secondary metabolites.** After comparison of the UPLC-HRMS data, the defatted organic layers for each condition were combined due to the similarity of their chemical profiles, so as to generate a larger pool of material for isolation studies, which were carried out using well-established natural products chemistry procedures (131, 132). The fractions were dissolved in CHCl_3_-MeOH, absorbed onto Celite 545 (Acros Organics), and fractioned by normal-phase flash chromatography using a gradient of hexane-CHCl_3_-MeOH. The isolation of the compounds was carried out using preparative high-performance LC (HPLC).

The isolated fungal metabolites were identified by direct comparison of the spectroscopic and spectrometric properties with those previously reported, and where possible, structures were validated by comparisons with authentic reference standards (133). Additionally, mass defect filtering (134) was used to identify structurally related analogs of the isolated compounds.

**(iv) General experimental procedures.** The nuclear magnetic resonance (NMR) data were collected using a JEOL ECS-500 spectrometer operating at 500 MHz for ^1^H and at 125 MHz for ^13^C, or an Agilent 700-MHz spectrometer, equipped with a cryoprobe, operating at 700 MHz for ^1^H and at 175 MHz for ^13^C. The HPLC separations were performed on a Varian Prostar HPLC system equipped with a Prostar 210 pump and a Prostar 335 photodiode array detector, with the collection and analysis of data using Galaxy Chromatography Workstation software. The columns used for separations were either a Synergi C_18_ preparative column (4 μm; 21.2 × 250 mm) at a flow rate of 21.2 mL/min, a Luna PFP(2) preparative column (5 μm; 21.2 × 250 mm) at a flow rate of 17 mL/min, or an Atlantis T3 C_18_ preparative column (5 μm; 19 × 250 mm) at a flow rate of 17 mL/min. Flash chromatography was performed on a Teledyne ISCO Combiflash Rf 200 as monitored by evaporative light-scattering and photodiode array detectors.

### Growth assays.

**(i) Antifungal drug susceptibility testing.** Antifungal susceptibility testing for both voriconazole (Sigma-Aldrich) and amphotericin B (Sigma-Aldrich) was performed by determining the MIC according to the protocol established by the Clinical and Laboratory Standards Institute.

**(ii) Stress response.** Radial growth was used to compare how the different strains responded to cell wall stress, iron starvation, and hypoxia. Strains were grown in solid minimal medium (MM), as described previously (135), inoculated with 1 × 10^5^ spores of each strain, and incubated for 5 days at 37°C before colony diameter was measured. To induce cell wall stress, 30 or 50 μg/mL of Congo red (cell wall perturbing) was added to the medium. For iron starvation, iron-poor MM was devoid of all iron and supplemented with 128 μg/mL of gallium nitrate (Sigma-Aldrich). Gallium is chemically similar to iron, and for this reason it is taken up by the cell to replace iron (136). Radial growth for the aforementioned stresses was expressed as a ratio, dividing colony radial diameter (in centimeters) of growth under the stress condition by the colony radial diameter in the control (no-stress) condition. For hypoxia analysis, the plates were incubated on 5% CO_2_ and 1% O_2_ at 37°C for 5 days.

Oxidative stress was measured using the protocol described by Canóvas et al. (137). Briefly, the experiments were performed in 96-well plates containing 100 μL of MM (with 1% agar) supplemented or not with 3 mM H_2_O_2_ (Merck S.A.) or 0.15 mM menadione (Sigma-Aldrich). Each well was inoculated with 1 × 10^5^ spores, and the growth was measured over time by quantifying the absorbance at 595 nm in a plate reader (Synergy HT) at 37°C. Data were recorded and analyzed with Gen5 data analysis software v2.0 and exported to Microsoft Excel for further analysis and generation of the graphs. Lag times were calculated using Gen5 data analysis software v2.0 as the time interval between the line of maximum slope of the propagation phase and the absorbance baseline at time zero.

**(iii) Statistical evaluation of growth assay results.** A one-way ANOVA was calculated for the iron starvation, hypoxia, and oxidative stress data sets, and a two-way ANOVA was calculated for the data set of cell wall stress with two Congo red concentrations. Graphs were visualized and statistics were calculated using GraphPad Prism v9.3.1.

### Assessment of virulence using an invertebrate model of fungal disease, Galleria mellonella.

**(i) Preparation of larvae and inoculum.** Galleria mellonella larvae were used to investigate the virulence of all seven strains. The larvae used for the infection were in the last larval stage of development (sixth week). All selected larvae weighed ~300 mg and were restricted to food for 24 h before the experiment. Fresh asexual spores (conidia) of each strain were counted using a hemocytometer.

**(ii) Inoculation and observation.** Five microliters of each inoculum was injected using a Hamilton syringe (model 7000.5 KH) through the last left ear (*n* = 10/group), resulting in 1 × 10^6^, 1 × 10^4^, and 1 × 10^3^ conidia/larva. The control group was inoculated with PBS. After infection, the larvae were kept with food restrictions at 37°C in petri dishes in the dark and scored daily for 15 days. The larvae were considered dead based on a lack of movement in response to touch. The viability of the inoculum administered was determined by serial dilution of the conidia in yeast extract-agar-glucose medium and incubating the plates at 37°C for 72 h. The experiment was repeated twice.

We separated and assembled the groups with the larvae (*n* = 10) in petri dishes. The groups were composed of larvae that were approximately 300 mg in weight and 2 cm long. Moth sex was not accounted for due to the impossibility of visually determining sex at the sixth week of larval development.

**(iii) Statistical analysis of infection rates.** Larval survival was plotted on Kaplan-Meier curves using the survival and survminer R packages. A Mantel-Cox log-rank test was used to evaluate statistical significance between the survival curves of larvae infected with different fungal strains.

### Assessment of virulence using a murine model of keratitis.

**(i) Preparation of fungal inoculum.** On the day of corneal inoculation, asexual spores (conidia) of *A. arachidicola* IC26646, A. flavus NRRL 1957, A. fumigatus Af293, and *A. parasiticus* NRRL 2999 were incubated in 25 mL yeast extract-peptone-dextrose broth at a density of 5 × 10^6^ conidia/mL at 35°C, 200 rpm. Once conidia were swollen and clumping but not polarized (approximately 4 h for all strains), cultures were collected by centrifugation, washed twice with PBS, resuspended in PBS to an optical density of 0.8 (360 nm), and stored at room temperature until the corneal inoculation (approximately 1 h).

**(ii) Corneal infections.** On the day preceding corneal inoculation, 6- to 8-week-old male C57BL/6J mice (Jackson Laboratories) were immunosuppressed with a 100 mg/kg methylprednisolone by intraperitoneal (i.p.) injection. The following day, animals were anesthetized with 100 mg/kg ketamine and 6.6 mg/kg xylazine, i.p., and the corneal epithelium was ulcerated over the pupil of the right eye to a diameter of ~1 mm by using an Algerbrush II. Five microliters of the fungal inoculum (described above) was pipetted over the ulcerated cornea and remained in place for 20 min before being removed with a Kim wipe. Five animals were included in each infection group, and the contralateral eye of each animal remained uninfected in accordance with the Association for Research in Vision and Ophthalmology guidelines for the use of animals in vision research. Animals were further injected subcutaneously with 1 mg/kg Buprenorphine SR for analgesia.

**(iii) Slit-lamp microscopy and disease scoring.** Each day postinoculation (p.i.), animals were anesthetized with isoflurane and imaged by slit-lamp using a Micron IV biomicroscope (Phoenix Technology Group, CA, USA). Images were deidentified and assigned an overall disease score (range, 0 to 4) by two blinded reviewers based on the area of opacification, density of opacification, and surface irregularity. The average disease scores for each cornea were compared by one-way ANOVA using GraphPad Prism v9.3.1.

**(iv) Optical coherence imaging and corneal thickness measurement.** Corneas were also imaged using the Bioptigen spectral domain-optical coherence tomography system (Leica Microsystems, Deerfield, IL, USA). Mice were anesthetized with isoflurane, and a 4- by 4-mm image was scanned with a 12-mm telecentric lens. Reference arm calibration was completed by the manufacturer and set to 885. Images were analyzed using the InVivoVue Diver software (Bioptigen). Briefly, corneal scans were digitally overlaid with a 11 by 11 spiderplot, and the distance between the epithelium and endothelium was measured at 11 distinct points near the central cornea. The average of the 11 measures was taken as the corneal thickness (in millimeters) and compared across groups using a one-way ANOVA in GraphPad Prism v9.3.1.

**(v) Fungal burden assessment.** At 72 h p.i., corneas were resected and homogenized by incubation in 1 mL of buffer containing 2 mg/mL collagenase I (Sigma) for 1 h at 37°C. Dilutions of the homogenate were plated onto inhibitory mold agar and incubated overnight at 35°C, and colonies were enumerated. Colony counts were compared between groups using a one-way ANOVA in GraphPad Prism v9.3.1.

### Data availability.

Sequencing data and genome assemblies associated with this project are compiled under BioProject PRJNA824811. Raw reads are available through the NCBI sequence read archive under accession numbers SRR19347655 (Aspergillus *arachidicola* IC26646), SRR19347656 (Aspergillus *arachidicola* IC26645), SRR19347505 (Aspergillus flavus NRRL 1957), SRR18725159 (Aspergillus flavus NRRL 501), SRR19347653 (Aspergillus parasiticus NRRL 2999), SRR19347654 (Aspergillus parasiticus NRRL 502), and SRR19369914 (Aspergillus *nomiae* NRRL 6108).

The NMR data for compounds **1**, **3**, **6**, **12**, **22**, **26** and **57** were deposited in the Natural Products Magnetic Resonance Database and can be accessed at https://npmrd-project.org/.

Supplementary tables and figures are available on FigShare at https://doi.org/10.6084/m9.figshare.20256336.
